# Evolution, types, and distribution of flight control devices on wings and elytra in bark beetles

**DOI:** 10.1038/s41598-024-57658-y

**Published:** 2024-03-24

**Authors:** Jakub Białkowski, Robert Rossa, Anna Ziemiakowicz, Jostein Gohli, Jakub Dymek, Jakub Goczał

**Affiliations:** 1 Department of Forest Ecosystems Protection, University of Agriculture in Krakow, 29 Listopada 54, 31-425 Kraków, Poland; 2 Division of Biotechnology and Plant Health, Norwegian Institute of Bioeconomy Research, Ås, Norway; 3 Department of Biology and Cell Imaging, Faculty of Biology, Institute of Zoology and Biomedical Research, Jagiellonian University, Kraków, Poland

**Keywords:** Evolutionary developmental biology, Evolutionary genetics, Phylogenetics, Speciation, Taxonomy, Entomology

## Abstract

Gaining the ability to fly actively was a ground-breaking moment in insect evolution, providing an unprecedented advantage over other arthropods. Nevertheless, active flight was a costly innovation, requiring the development of wings and flight muscles, the provision of sufficient energetic resources, and a complex flight control system. Although wings, flight muscles, and the energetic budget of insects have been intensively studied in the last decades, almost nothing is known regarding the flight-control devices of many crucial insect groups, especially beetles (Coleoptera). Here, we conducted a phylogenetic-informed analysis of flight-related mechanosensors in 28 species of bark beetles (Curculionidae: Scolytinae, Platypodinae), an economically and ecologically important group of insects characterized by striking differences in dispersal abilities. The results indicated that beetle flight apparatus is equipped with different functional types of mechanosensors, including strain- and flow-encoding sensilla. We found a strong effect of allometry on the number of mechanosensors, while no effect of relative wing size (a proxy of flight investment) was identified. Our study constitutes the first step to understanding the drivers and constraints of the evolution of flight-control devices in Coleoptera, including bark beetles. More research, including a quantitative neuroanatomical analysis of beetle wings, should be conducted in the future.

## Introduction

Gaining the ability to fly actively was a ground-breaking moment in insect evolution. First pterygote lineages hit the air probably in the early Carboniferous, about 90 million years before it was done by the first vertebrates^1^. The remarkably rapid diversification of Insecta, manifested in the formation of more than fifteen orders by the end of the Carboniferous^2,3^, exemplifies the adaptive importance of this innovation. Powered flight allows insects to escape predators, overcome migration barriers, exploit novel resources, and find a mate. Nevertheless, active flight is also a physiologically and developmentally costly investment, as it requires the development of an effective flight apparatus (wings and muscles), the provision of sufficient energetic resources, and the formation of a complex flight control system. Although the first two elements have been intensively investigated in the last decade, even basic information on the functioning of flight-related sensory feedback is still missing for most insect groups.

The control system of an insect’s active flight has two major sensory modalities: vision and mechanosensation, supported by additional sources of input (e.g., hemosensory or thermosensory)^4^. For a long time, vision was considered a major and sufficient source of input for flying insects, allowing them to navigate in complex environments^5^. Nevertheless, more recent studies have indicated that insects’ vision has numerous limitations, including especially long processing delay that might result in instability when operating during fast flight or rapid acceleration^5,6^, and significantly reduced efficiency in poor lighting conditions^5,7^. These discoveries have pushed scientists to study the role and functioning of other sensory inputs that are potentially important for flight coordination^5,7^. A growing body of evidence now points to the role of mechanosensation as a highly important modality required for effective course control during the aerial maneuvers of insects^4,5,8–11^. Specialized mechanoreceptors located on flight apparatuses (either on the surface of wings or on their modified forms such as halteres or elytra) transduce local strain fluctuations and airflow pressure into neural signals, providing extremely fast feedback regarding airflow and aeroelastic deformations of the wings surface, which is critical for effective flight control^4,8,11^. Unfortunately, despite the recent progress in our understanding of the functional diversity of wing mechanosensors^4,8,11^, even basic information regarding the morphology, distribution, and evolution of these structures is still lacking for many key taxonomic groups of insects^4,8,11^.

Our knowledge on the flight-control devices is particularly poor in the order Coleoptera (beetles). Although beetles account for about one fifth of all animal species on our planet^12^ and exhibit spectacular flight apparatus modification (transformation of fore wings into hardened elytra, together with the development of a sophisticated hind wing folding mechanism), all current knowledge on flight-related mechanosensors located on wings in this order is confined to a morphological analysis of a single species *Dytiscus marginalis* Linnaeus (Dytiscidae) from the first half of the twentieth century^13,14^. Although the distribution of flight-related mechanosensors on beetles' elytra has been recently investigated morphologically in several species (but not in bark beetles)^10,15,16^, existing studies have not taken into account the phylogenetic background and were thus not suited for evaluating mechanosensor diversity patterns across larger monophyletic group of beetles.

Within the order Coleoptera, bark beetles (s*ensu lato*) (Curculionidae: Scolytinae, Platypodinae) encompass some of the most spectacular dispersers. These small insects can use both active and passive (aided by the wind) flight to disperse over impressive distances of more than 100 km per day (via passive flight)^17^. Moreover, bark beetles exhibit extreme within- and between-species variation in dispersal capacity; ranging from no-flyers to individuals conducting exceptionally long flights within the same sex and population^18^. They have also developed sophisticated host-finding strategies^19^ and co-colonizing strategies^20^. Scolytids are also an interesting illustration of the dispersal-reproduction trade-off phenomenon, with inbreeding or partially inbreeding species, dispersal polymorphism, and an ability to degenerate flight muscles to regain energy^21–23^. This, together with the unprecedented economic and ecological importance of the group^22,24^, makes bark beetles an interesting taxa for studying the evolution of flight control devices.

The first, crucial step to understanding the flight control system of bark beetles is to characterize their wing mechanosensors, especially their structural diversity, distribution, and number. In the present study, we use such data to address the following questions: (1) What types of wing mechanosensors are distributed on the wings and elytra of bark beetles? (2) How many mechanosensors are on the wing, and where are they located? (3) Does the number of mechanosensors increase as body size increases? (4) Is the number of mechanosensors related to the level of flight investment (relative wing size)? (5) Can the differences in the number of sensors be fully explained by phylogeny?

We believe that answering this question will not only contribute to a better understanding of the flight control system in bark beetles but also advance our knowledge regarding the design of information-gathering devices used by insects for controlling of active flight.

## Results

### Types, distribution and number of mechanosensors

**Figure 1:**
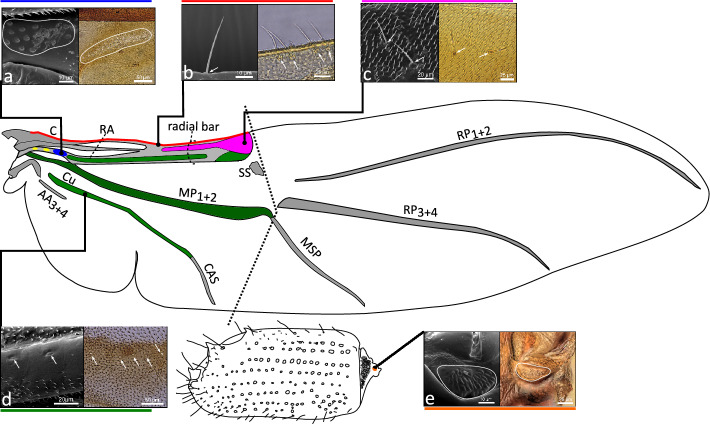
Distribution of various types of flight-related mechanosensors on hind wings and elytra of bark beetles: (**a**) campaniform sensilla fields, (**b**) wing margin trichoid sensilla, (**c**) isolated trichoid sensilla, (**d**) isolated campaniform sensilla, (**e**) Lehr’s field (campaniform sensilla field located on the root of elytra). SEM images are given on the left side, and optical microscope images are given on the right side of each magnified picture. The dotted line indicates transverse wing fold. Yellow areas indicate the location of two small MS fields that have not been analyzed (see “Materials and Methods”). Other colored areas indicate regions where a particular type of mechanosensor might be distributed. Abbreviations: C—Costal edge (leading edge), RA—Radius Anterior, Cu—Cubitus/Cubital Vein, AA_3+4_—Anal Anterior (branches 3 and 4), CAS—Cubitoanal Strut (Cu + CuP + AA_3_), MP_1+2_—Media Posterior (branches 1 and 2), MSP—Medial Spur (continuation of MP_1+2_), SS—Support Sclerite, MJ—Marginal Joint, RP_3+4_—Radius Posterior (branches 3 and 4), RP_1+2_—Radius Posterior (branches 1 and 2).

**Figure 2:**
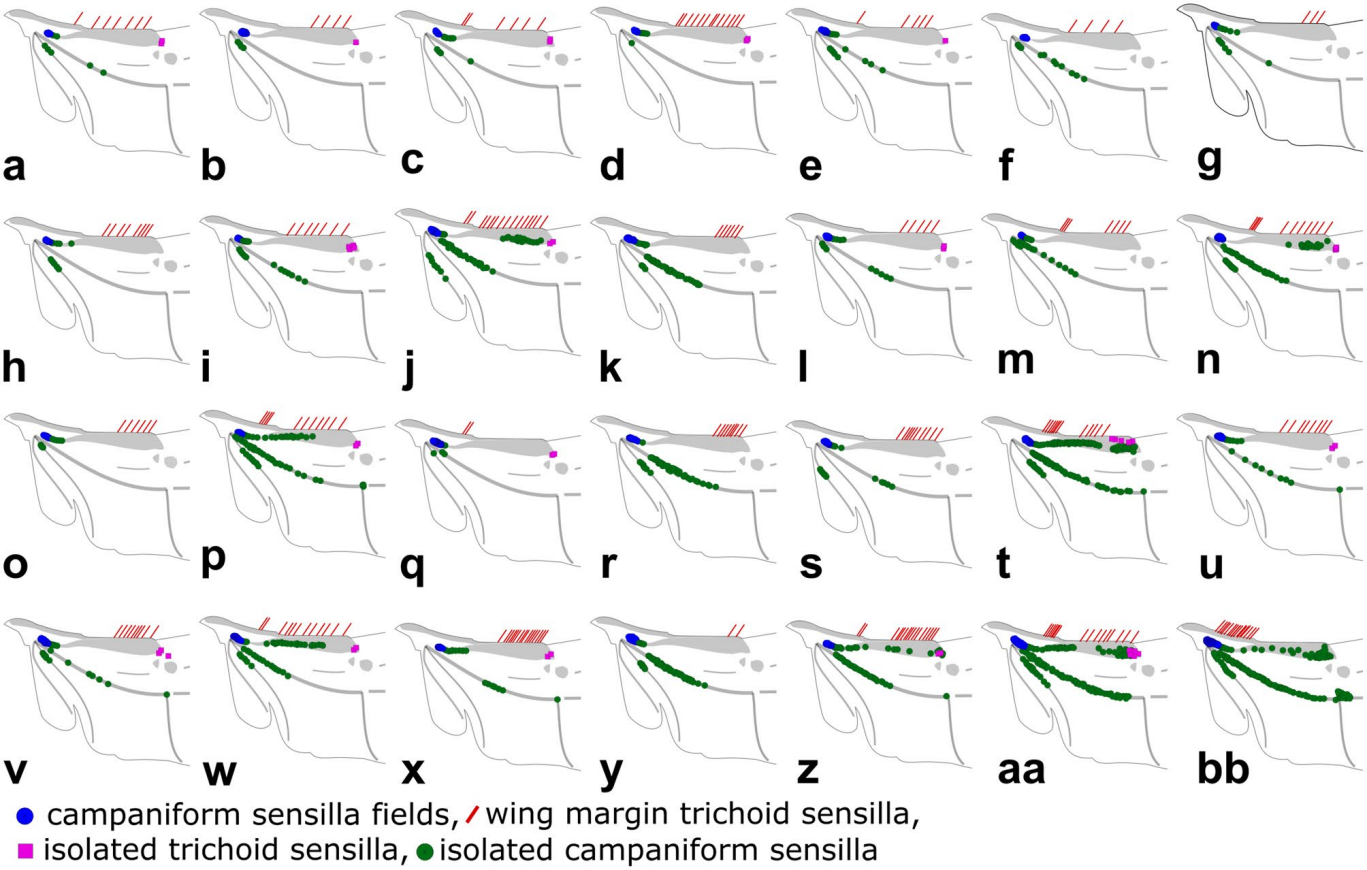
Distribution of various types of flight-related mechanosensors on hind wings of studied species. Species are ranked from smallest to largest based on the wing area: (**a**) *Crypturgus cinereus*, (**b**) *Pityophthorus pityographus*, (**c**) *Ernoporus tiliae*, (**d**) *Cryphalus piceae*, (**e**) *Xyleborinus saxesenii*, (**f**) *Pityogenes chalcographus*, (**g**) *Dactylotrypes longicollis*, (**h**) *Hylurgops palliatus*, (**i**) *Dryocetes alni*, (**j**) *Phloesinus thujae*, (**k**) *Scolytus rugulosus*, (**l**) *Pityokteines vorontzowi*, (**m**) *Taphrorychus bicolor*, (**n**) *Polygraphus poligraphus*, (**o**) *Gnathotrichus materiarius*, (**p**) *Hylastes opacus*, (**q**) *Platypus cylindricus*, (**r**) *Scolytus intricatus*, (**s**) *Anisandrus dispar* (female), (**t**) *Hylesinus fraxini*, (**u**) *Ips acuminatus*, (**v**) *Orthotomicus laricis*, (**w**) *Trypodendron domesticum*, (**x**) *Ips cembrae*, (**y**) *Scolytus ratzeburgii*, (**z**) *Ips sexdentatus*, (**aa**) *Dendroctonus micans*, (**bb**) *Dactylipalpus* sp.

Based on differences in morphology and structural arrangement, we have identified four major types of wing mechanosensors on the dorsal side of hind wings of the studied bark beetle species: campaniform sensilla fields (Figs. 1a, 2a–bb), wing margin trichoid sensilla (Figs. 1b, 2a–bb), isolated trichoid sensilla (Figs. 1c, 2a–bb), and isolated campaniform sensilla (Figs. 1d, 2). It should be mentioned here, that in some other studies trichoid sensilla are sometimes called bristles or bristled sensilla^11,25^.

All types of mechanosensors were distributed only within the proximal part, and no sensilla were identified on the distal part of a wing (within folded apical field) behind the transverse wing folding line (Fig. 1—dotted line). Wing margin trichoid sensilla (2 min, 9 avg., 26 max) were distributed along the front-most (leading) edge of the wing (costal edge), beginning from the wing base and reaching the end of the radial bar (Fig. 2a–bb). Campaniform sensilla fields (three fields, but only the last, most distant field was analyzed—see “Materials and Methods” section) were located at the base of the radial anterior vein (Fig. 2a–bb). Analyzed sensilla fields were composed of different numbers of campaniform sensilla (4 min, 31 avg., 161 max). For the vast majority of the analyzed species, isolated trichoid sensilla (0 min, 2 avg., 11 max) were situated at the expanded end of the radial bar and only in rare cases were more or less regularly scattered over the distant part or the radial vein (Fig. 2a–bb). Isolated campaniform sensilla were found in different regions of wing venation (Fig. 2a–bb) including, cubitus/cubital vein (0 min, 5 avg., 18 max), media posterior vein (0 min, 17 avg., 100 max), end of the media posterior vein (0 min, 2 avg., 41 max), or radial vein (including radial bar) (0 min, 18 avg., 130 max). While wing margin trichoid sensilla, campaniform sensilla fields, and isolated campaniform sensilla were present in every studied genus, isolated trichoid sensilla were found in 18 out of 28 analyzed genera.

On the dorsal side of each elytron, a single campaniform sensilla field (called Lehr’s field^10^; Fig. 1e) was identified. Kidney-shaped fields were always located on the elytra root (Fig. 1e). Lehr’s fields differed in area (126 µm^2^ min, 1449 µm^2^ avg., 19,542 µm^2^ max), and were composed of different numbers of campaniform sensilla (8 min, 26 avg., 98 max).

The included species differed significantly in their total number of mechanosensors on hind wings (Kruskal–Wallis χ^2^ = 116.00; *P* < 0.001), but there was no difference in the MS number between males and females [(Mann–Whitney U test, U = 1432.5, *P* > 0.01, *Dactylipalpus* sp. (no males available), and *Anisandrus dispar* (males with reduced hind wings) were excluded priori the analysis)]. The post-hoc Dunn's test based on a Bonferroni^26^ corrected alpha indicated that 14 pairs of species differed significantly in the total number of mechanosensors on wings (*P* < 0.05). *Cryphalus piceae* differed from *Dactylipalpus sp*. *C. cinereus* differed from *Dactylipalpus sp*., *D. micans*, *H. fraxini* and *S. ratzeburgii*. *Dactylipalpus sp*. differed from *D. longicollis*, *E. tiliae*, *P. chalcographus* and *P. pityographus*. *Dactylotrypes longicollis* differed from *D. micans* and *H. fraxini*. *Dendroctonus micans* differed from *P. pityographus*. *Hylesinus fraxini* differed from *P. pityographus*. *Pityophthorus pityographus* differed from *S. ratzeburgii*. The post-hoc test detected significant differences in mechanosensor numbers on hind wings only among selected pairs of species. This might be attributed to the low sample size and weak statistical power of the non-parametric post-hoc tests (when compared to parametric approaches)^26^.

We found significant differences in the total number of mechanosensors on elytra (Kruskal–Wallis χ^2^ = 109.97; *P* < 0.001) among species, and also in area of the sensing field (Lehr’s field) on elytra (Kruskal–Wallis χ^2^ = 105.00; *P* < 0.001). The post-hoc Dunn's test based on a Bonferroni^26^ corrected alpha indicated that 18 pairs of species differed significantly in the total number of mechanosensors on wings (*P* < 0.05). *C cinereus* differed from *Dactylipalpus sp*., *D. micans*, *H. fraxini*, *P. thujae* and *S. ratzeburgii*. *Dactylipalpus sp.* differed from *D. alni*, *P. chalcographus*, *P. vorontzovi*, *P. pityographus* and *T. bicolor*. *Dendroctonus micans* differed from *P. vorontzovi* and *P. pityographus*. *Dryocetes alni* differed from *P. thujae*. *Hylesinus fraxini* differed from *P. vorontzovi* and *P. pityographus*. *Phloesinus thujae* differed from *P. vorontzovi*, *P. pityographus* and *T. bicolor*. There was no difference in the total number of mechanosensors on elytra, nor in the Lehr’s field area between males and females (Mann–Whitney U test, U = 1406.05, *P* > 0.01 and U = 1388.50, *P* > 0.01, respectively). *Dactylipalpus* sp. (no males available), and *Anisandrus dispar* (males with reduced hind wings) were excluded from this analysis.

### Effect of phylogeny, flight investment and allometry on mechanosensors number

**Figure 3:**
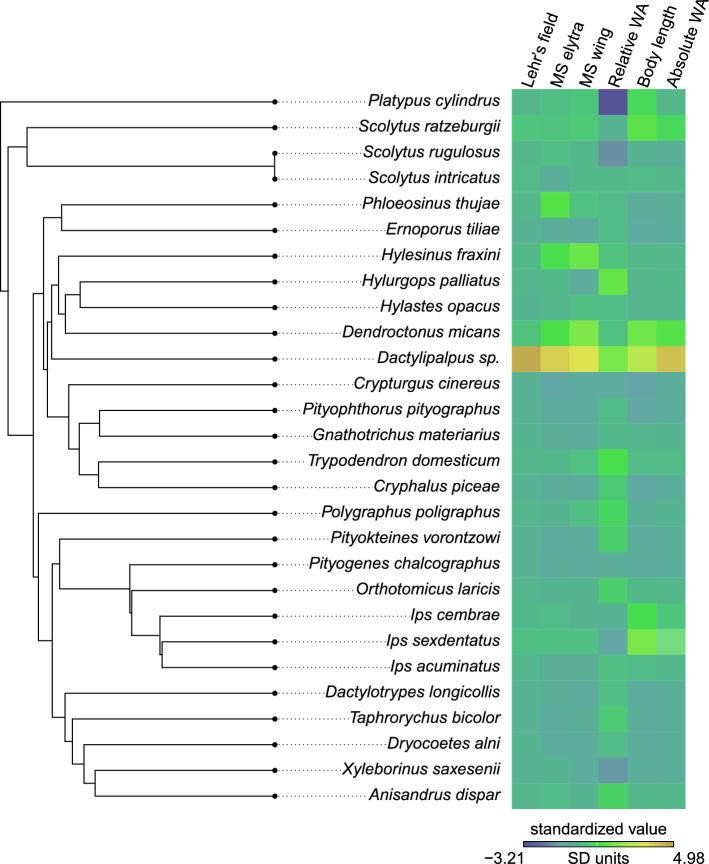
The phylogeny used in the phylogenetic comparative analyses. The heatmap shows species averaged values for the predictor and response variables used in our SLOUCH analyses. The data was standardized (distance to the cross-species mean in standard deviations) to enable mapping on a single colour scale. MS denotes number of mechanosensors, while WA is wing area.

We observed a weak phylogenetic signal in the total number of mechanosensors on elytra, and in the area of Lehr’s field on elytra (Fig. 3). There was a stronger phylogenetic signal for the total number of mechanosensors on hind wings (Fig. 3). These results were consistent across the datasets with and without the outlier *Dactylipalpus sp.* (Table 1, Supplementary Information S1—Table S1).

**Table 1:** Results from SLOUCH models where our measure of flight investment (relative wing area, i.e., residuals from a linear model where body length was regressed on wing area), body length and absolute wing area was regressed on mechanosensor response variables. Here, one outlier species (*Dactylipalpus* sp.) was removed from the analysis. Abbreviations: MS—mechanosensors, WA—wing area. The model parameter values correspond to SLOUCH models where phylogenetic inertia has been controlled for, i.e., the optimal regressions.

Response	Predictor	n	Intercept only	Phylogenetic half life	Stationary variance	R^2^	AICc	δAICc*
Total number of MS on elytra	Relative WA	27	0.000	0.000	352	0.01	245	2.63
	Body length	27		0.000	250	0.29	236	− **6.62**
	Absolute WA	27		0.000	251	0.29	236	−** 6.54**
Lehr's field area on elytra	Relative WA	27	0.003	0.214	0	0.53	472	3.07***
	Body length	27		0.004	4.E + 05	0.76	433	− **35.83**
	Absolute WA	27		0.034	0	0.84	428	− 41.42***
Total number of MS on hind wings	Relative WA	27	0.141	171	2.E + 06	0.21	333	− 0.71**
	Body length	27		113	4.E + 05	0.47	321	− 13.04**
	Absolute WA	27		0.025	6112	0.42	323	− **11.11**

*AICc values from SLOUCH models with relative wing size compared to null models. δAICc values < − 2 are considered significant. **Models with uncertain estimation (no clearly defined likelihood peak) of half life and stationary variance. ***Models with uncertain estimation (no clearly defined likelihood peak) of stationary variance.

**Figure 4:**
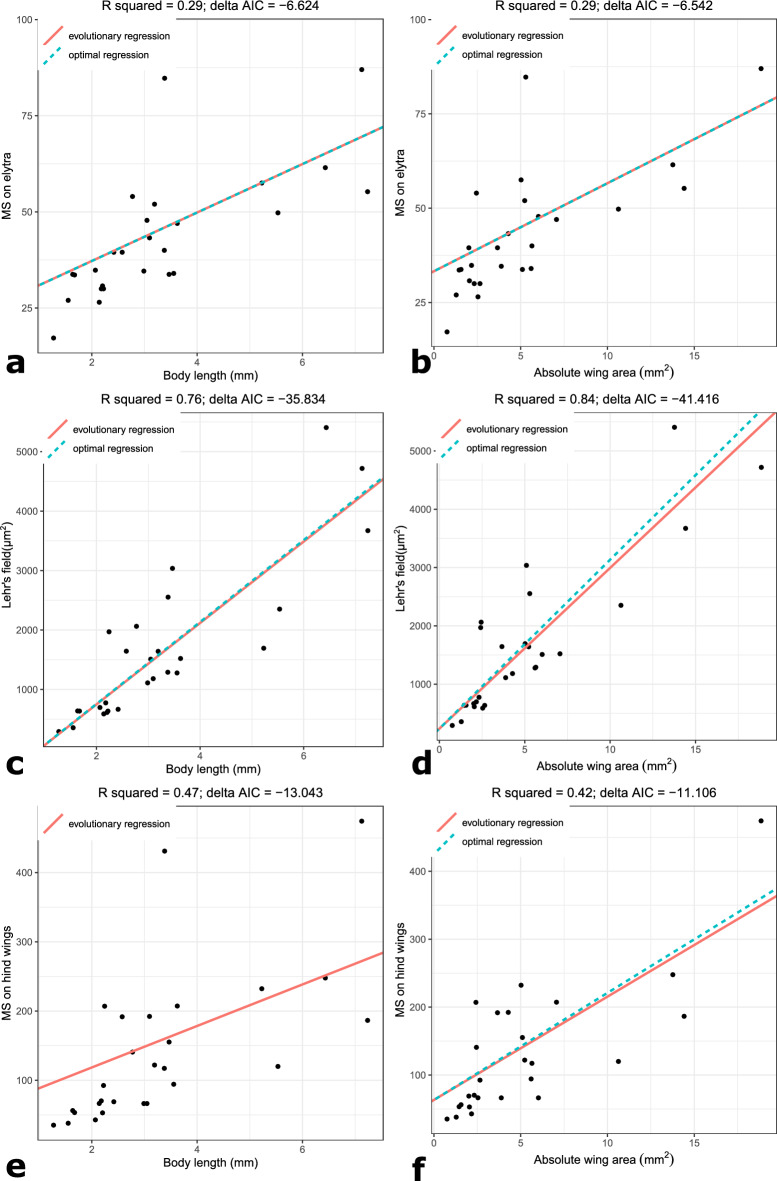
Effect of selected variables on the number of mechanosensors on elytra and hind wing and on the area of Lehr’s field: (**a**) Body length regressed on the total number of mechanosensors on elytra, (**b**) Absolute wing area regressed on the total number of mechanosensors on elytra, (**c**) Body length regressed on the Lehr’s field area, (**d**) Absolute wing area regressed on the Lehr’s field area, (**e**) Body length regressed on the total number of mechanosensors on hind wings, (**f**) Absolute wing area regressed on the total number of mechanosensors on hind wings. The evolutionary regression shows the observed association, while the optimal regression shows the effect size expected in a scenario with no phylogenetic inertia (evolutionary lag). One outlier (*Dactylipalpus* sp.) was removed from these analyses; the analyses of the entire data set is available in the Supplementary Information S1—Table S1, Fig. S1. The optimal regression is not shown for the regression of body length on the total number of mechanosensors on hind wings, due to uncertain estimation of the half-life parameter. R squared denotes the amount of variation in the response explained by the model, while delta AIC gives the significance level, with values < − 2 being considered statistically significant.

We found no statistical support for flight investment, using relative wing size as a proxy, being associated with the number of mechanosensors nor with the size of Lehr’s field (Table 1, Supplementary Information S1—Tables S1, S2, S3). We did observe a strong association between both body length and absolute wing size and the response variables (Fig. 4a–f, Table 1), but note that body length and absolute wing size were found to be strongly correlated (Supplementary Information S1—Fig. S2). These results were consistent across the two datasets that either included or excluded *Dactylipalpus* sp. (Table 1, Supplementary Information S1—Tables S1, S2, S3), although the full dataset had stronger δAICc values (Supplementary Information S1—Tables S1, S3).

Across all performed analyses, body length and absolute wing area were significant predictors, also when analysing the number of campaniform and trichoid sensilla separately (Supplementary Information S1—Tables S2, S3). The one exception was for isolated trichoid sensilla, which was not significantly associated with neither body length nor absolute wing area. Relative wing area was not a significant predictor in any of the performed tests. We note that some tests did not converge on clearly defined likelihood peaks (Supplementary Information S1—Tables S2, S3), which means that the way in which SLOUCH controlled for phylogenetic inertia is unreliable, causing regression slope estimates to be similarly unreliable. However, several tests converged well, and the overall results from all performed analyses clearly indicate that body length (or absolute wing area) is important in the context of mechanosensor diversity patterns across the included species.

Generally, there was little evidence for substantial evolutionary lag on the statistically significant associations found (Fig. 4a–f). The largest half-life estimates, and thereby the biggest difference in slope between evolutionary and optimal regression lines, were observed in analyses of the dataset which included the outlier *Dactylipalpus sp.* (Supplementary Information S1—Tables S1, S3), particularly the test of body length against Lehr’s field.

For some models, the estimation of half-life or stationary variance was not clear, i.e., there was no defined peak in the likelihood landscape of the parameter estimation. These cases are provided in the Supplementary Information S1—Tables S2, S3. Uncertainty surrounding the half-life estimation is problematic (the stationary variance less so, as it simply denotes the residual variance) due to its impact on the effect size of the optimal regression. In the model where body length was regressed on the total number of mechanosensors on hind wings, SLOUCH did not return a clearly defined likelihood peak for the half-life parameter (Supplementary Information S1) and hence we did not include the optimal regression line for this association in Fig. 4e. Apart from this model, the half-life parametrization was only uncertain in non-significant models with relative wing size as the predictor.

## Discussion

For the first time, we have conducted across-species phylogenetic-informed analysis of flight-related mechanosensors in the order Coleoptera. The results indicated that beetle hind wings are equipped with different types of mechanosensors, including: campaniform sensilla fields, wing margin trichoid sensilla, isolated trichoid sensilla, and isolated campaniform sensilla. Whereas, on the modified fore wings (elytra) only one type of mechanosensor – campaniform sensilla field (or Lehr’s field) was identified. In some studies, mechanosensors located on the wings of other insects groups are subdivided into additional subgroups based on their spatial arrangement (e.g.,^11^) but we think that it might be subjective and controversial, without a quantitative neuroanatomical analysis, and thus we decided to group them only into basic morphological categories. From the functional perspective, identified mechanosensors can be subdivided into two groups^11^: strain-encoding sensilla (campaniform sensilla fields, Lehr’s fields, isolated campaniform sensilla), and flow-encoding sensilla (wing margin trichoid sensilla, isolated trichoid sensilla). Flight-related sensilla were located on different regions of the flight apparatus, including the root of the elytra, the leading edge of the wing and several main veins (e.g., radial, medial, cubitus) of the hind wings. Mechanosensors on hind wings were identified on the proximal region of wings only; no sensing structures were found behind the transverse folding line (on the apical field). The number of mechanosensors on wings and elytra varied significantly among the studied bark beetle species, and the observed differences can be largely explained by allometry. In general, larger species have a proportionally higher number of mechanosensors on wings and elytra (both trichoid and campaniform), and no effect of flight investment (relative wing size) on the mechanosensor number was found.

The observed patterns correspond well with some of the earlier described general trends in the spatial distribution of sensilla on insects’ wings (see^8^). Like in other insect groups, mechanosensors are located on both fore wings (here transformed into elytra), and hind wings. On the latter, sensilla are distributed only on the main wing veins and within the leading edge of the wing. The most proximal wing mechanosensors are always of campaniform type and form elliptical fields concentrated towards the leading edge of the wing^8^. Importantly, the total number of mechanosensors on wings exhibits a clear positive correlation with wing size, as was also observed in other insect orders^8^. Nevertheless, the number of mechanosensors on modified fore wings (elytra) is clearly lower than the number of mechanosensors on hind wings, which is the opposite pattern to that most commonly seen in most insects^8^. In the vast majority of insect species studied to date, mechanosensors were more abundant on the proximal part of the wing than on the distal part^8^. The species studied here seem to be an extreme example of this pattern, as no sensing structures were identified at all on the distal part of the wing (behind the line of transverse wing folding). Beetles have evolved unique transverse wing fold mechanisms, enabling secure storage of hind wings beneath the elytra when not in use^27^. This resulted in significant modifications to the original wing venation, for instance, the formation of elastic features that enabled the transverse fold of the wing blade. Wing structures are thus thinner and more flexible at the bending zones, which might potentially constraint the distribution of tracheolae and wing nerves. More beetle species from different evolutionary lineages have to be analyzed in the future to verify this hypothesis.

Relative wing size is often used as a proxy for the level of selective pressure on flight abilities in insects^28–30^, and bark beetles are considered to be a group with a high between-species variation in dispersal abilities^20^. Nevertheless, we found no support for the hypothesis assuming that the species with a larger relative wing size (investing more resources in flight wing development) have more flight-related mechanosensors on their flight apparatus. Nevertheless, dwarf and flightless males of *Anisandrus dispar* (Fabr.) were characterized by a clearly lower number of mechanosensors on both wings (14—mean for males, 61—mean for females) and elytra (17—mean for males, 26—mean for females) than their macroelytrous flight-capable females. This suggests that the loss of flight abilities results in decrease of flight-related mechanosensors number on the flight apparatus. Similar results were found for other non-flying beetles in studies on flight-related mechanosensors on elytra^16^. On the other hand, it has to be emphasized that relative wing size is only a simple approximation of dispersal potential. The actual flight capacity of bark beetles is a complex characteristic that depends on various factors, including wing size and shape, flight muscle volume or fat content^20^. Nevertheless, comprehensive data regarding the actual flight capacity of Scolytinae is available only for a few species, and their applicability in evolutionary studies is largely limited due to significant methodological discrepancies (e.g., flight mill vs. mark-recapture experiments).

Functional interpretation of the described patterns of mechanosensor distribution on bark beetle wings and elytra is challenging due to a lack of neuroanatomical research focused on wing mechanosensors in the order Coleoptera. However, the morphological compatibility of the described sensing structures with those identified in other insect groups, as well as similarity in the general aerodynamics of insect flight, allow us to formulate certain hypotheses. All insects lack muscles in their wings, and thus, active adjusting of the shape of the wing blade is more difficult than in birds or bats. To overcome this issue, insect wings elastically deform (bend and twist) their surface during flapping as a result of the inertial and aerodynamic forces generated by the flapping motion^31–34^. While various groups of thoracic flight muscles control wingbeat kinematics, the mechanical characteristics of the elastic wing structure accomplish a large portion of the change in wing blade twist and camber within each flapping cycle^31,35,36^. This requires precise feedback regarding the level of airflow and local wing blade deformations, resulting in specific patterns of mechanosensor distribution on insect wings.

In the studied bark beetle species, a series of trichoid sensilla were identified within the leading edge of the wings, which seems to be a common pattern in insects^11^. The front-most edge of the insect wing plays a crucial role in flight, as a vortex is typically generated through air flow separation at the leading edge. A growing body of evidence indicates that the leading-edge vortex plays one of the most important roles in an unsteady mechanism for high lift force generation in flapping wings^37–39^. In turn, the wing leading edge is exposed to large fluctuating pressures^40–43^ characterized by periodic changes in the airflow direction^11^. It has been hypothesized that trichoid sensilla distributed along the leading edge might therefore be involved in detecting the timing and intensity of vortex formation and shedding^11^. Empirical evidence is still scarce, but neuroanatomical studies on silk moth support this hypothesis, showing that trichoid sensilla located on the wing margin exhibit clear directional sensitivity to oscillating, but not to constant airflow^25^. In the studied bark beetle species, isolated trichoid sensilla were also found on the radial bar—a thick longitudinal structure reinforcing the leading edge of the wings which was formed by the fusion of subcosta posterior and radial veins^44^. The function of the trichoid sensilla located on the radial bar is unclear. It might be speculated that localization of flow-sensing devices in the immediate vicinity of the transverse wing folding point (apical hinge) might be potentially responsible for detecting extreme airflow pressure, whose occurrence could result in undesirable bending of the apical part of the wing.

Campaniform sensilla were the dominant type of mechanosensor on bark beetle wings. Different forms of their spatial arrangement (clustered-forming sensing fields, distributed linearly with relatively regular intervals, or randomly scattered) are most likely linked to their different functional properties. In all insect orders studied today, campaniform sensilla fields have been found at the wing base^8^, which seems to be a conserved feature among the whole Pterygota. Such fields are most likely responsible for detecting body rotations^9,45–47^, and various spatial arrangements and orientations of CS within the fields allow for the detection of different axes of rotation^48^. Moreover, it was hypothesized that campaniform sensilla fields located at the wing base might also be involved in the inertial sensing and/or tracking of wing loading^11^. CS in bark beetles are also distributed within several main veins, and their range sometimes reaches up to the end of the radial and medial veins. It was shown that campaniform sensilla distributed along wing veins in the blowfly were sensitive to both dorsal and ventral chord-wise deflections of the wing blade^49^. Moreover, comprehensive structural analysis of dragonflies' wings has shown that inertial loading exhibits strain field propagation along the main wing veins, which has stereotyped spatial profiles^11^. Campaniform sensilla distributed along the main wing veins are thus most likely involved in the detection of local deformations, occurring in different wingbeat phases^8^. Finally, isolated and scattered CS located near the transverse wing folding lines might be involved in monitoring extreme deformations, which is crucial for preventing unwanted folding of the apical field during flight.

The potential function of the campaniform sensilla field (Lehr's field) located on the elytral root was discussed by Frantsevich et al.^10^. Although the elytra of beetles do not play an active role in flight, they indirectly affect lift force and flight stability^50^. Except for rare exceptions, beetles widely spread their elytra before and during flight to release and unfurl their hind wings. During active flight, spread elytra experience significant air pressure, which is transferred to their articulation (root). It is thus highly likely that Lehr’s fields on elytra transduce strain experienced by the elytra bases when elytra are open during flight^10^. Although this hypothesis has never been empirically tested, the direct relationship between Lehr’s field development and flight capacity is best confirmed by the fact that these structures are reduced in flightless beetle species^16^.

From the evolutionary perspective, the obtained results suggest that natural selection leads to very rapid changes in both Lehr’s field area and the number of sensilla within the field on beetle elytra, whereas a certain level of phylogenetic inertia was found in the mechanosensor number on hind wings. Beetle elytra are highly modified and largely simplified fore wings, with significant homologies among unrelated beetle species^50^, while hind wings are more complex structures exhibiting high evolutionary stasis^44,51^. It seems that the evolution of mechanosensors on hind wings in bark beetles is therefore constrained by wing morphology, especially wing venation. Our results suggest that, due to the sensing limitation of a particular sensilium, the number of mechanosensors on flight apparatus has to increase proportionately with body and wing size to ensure proper coverage of sensory feedback, but only if other key wing characteristics (e.g., wing venation) remain the same. More research is needed in the future to fully understand drivers and constraints of the evolution of flight-control devices in Coleoptera, including bark beetles. In particular, a quantitative neuroanatomy survey of beetle flight wings is highly desirable, as it would allow for understanding the role of various mechanosensors' types in neural routing.

## Materials and methods

### Studied taxa

In total, 120 specimens of 28 species, representing 24 genera, were included in our study (Supplementary Information S1—Data S1). When possible, equal numbers of males and females for each species were included (Supplementary Information S1—Data S1). Specimens were randomly selected from the insect collection at the Department of Forest Ecosystem Protection, Faculty of Forestry, University of Agriculture in Krakow.

### Sample preparation and morphological analysis

All individuals were identified to species and sex^52,53^, after which specimens were photographed using a Keyence VHX-7000 4K high accuracy digital microscope (Keyence, Japan). Subsequently, both right and left elytra and wings were carefully detached from the body using micro surgical instruments, cleaned with absolute alcohol and rinsed in distilled water. Elytra were mounted on a small piece of mounting putty and placed on microscope slides (flexibility of mounting putty allowed for precise positioning of elytra). Hind wings were placed on microscope slides, strengthened using a preparation needle and small brush, coated with a thin layer of Euparal (synthetic microscopy mountant; Carl Roth GmbH, Germany) and covered with a cover slip. Wing and elytra preparations were subsequently photographed using Keyence VHX-7000 microscope.

Body length [mm], hind wing area [mm^2^], elytra area [mm^2^], Lehr’s field area [µm^2^] (campaniform sensilla field located on the elytra root—see^10^) were measured using Digimizer v. 6.3.0 software (MedCalc Software Ltd, Belgium; https://www.digimizer.com/). The number of mechanosensors (both campaniform and trichoid sensilla) were counted for each specimen on left and right wing (separately for each wing vein), and on right and left elytra. In order to provide high accuracy in the mechanosensor count, it was performed independently by two operators (JB and AZ), who manually inspected each wing preparation at different focus levels using a Keyence VHX-7000 microscope. If the results were unequal, SEM images were obtained to verify the mechanosensors counting. For SEM imaging, material was placed onto aluminium holders, covered by Leit-C (Sigma-Aldrich), sputtered with gold using JEOL JFC-1100E, and analysed using a JEOL JSM5410 scanning electron microscope. The following parameters were used for SEM image capture: magnification × 200, × 350, × 500; accelerating voltage 15 kV; working distance 12 mm.

Given that even basic information regarding flight capacity is currently unavailable for the vast majority of bark beetles, including almost all studied species, we decided to use an indirect approximation of the strength of selective pressure on flight apparatus development. For this reason, we calculated the body-size independent variable named ‘relative wing size’^54,55^ using residuals from log-transformed body length regressed on log-transformed wing area. Numerous studies show that wing size in insects is positively related to flight frequency, speed, and duration^56–59^. For instance, a cross-order analysis based on 102 insect species showed that the use of wing area provides the best prediction of actual insect wingbeat frequency^60^. Wing size has often been used as a proxy for dispersal capacity in insects, for instance in studies of the macroecological patterns of *Drosophila* flies^28^, rapid expansion of speckled wood butterfly^54^, evolution of flight morphology in stick insects^30^, prediction of range size and site occupancy in *Enallagma* damselflies^61^, estimation of dispersal potential and related range size in stoneflies^62^, a meta-analysis of the traits affecting dispersal ability in butterflies^63^ and the trade-offs between horn size and flight capacity in a rhinoceros beetle^64^. It has also been shown that wing area is one of the most important morphological factors that influence the actual flight capacity of the bark beetle—*Dendroctonus ponderosae* Hopkins^29^.

In order to analyze differences in the number of mechanosensors on the wings and elytra of the studied species, non-parametric tests (Kruskal–Wallis rank test, Mann–Whitney U test) were conducted.

### Phylogenetic reconstruction

A genera level phylogenetic topology was obtained from the literature, starting with the phylogeny published by Gohli et al.^65^, which was based on five genes (CO1, EF-1a, 28S, CAD, ArgK). All genera not represented in our data set was pruned from the Gohli et al. phylogeny, before missing genera were coded into this pruned tree based on the phylogeny published by Pistone et al.^66^. The genera *Ips* and *Scolytus* had more than one species representative; these were manually coded into the tree based on other published phylogenies^67,68^.

Node heights of the phylogeny were inferred by analysing COI sequences downloaded from BOLD in BEAST v2.7.3.^69^. The topology was not sampled, i.e., the topology from the literature-based phylogeny was fixed during the analysis. Per ModelTest-NG^70^ we used a GTR site model, with gamma category count set to four and included estimation of invariant sites. The analysis was performed with a Yule tree prior, and a strict clock.

### Phylogenetic comparative analyses

For testing the hypothesis that the number of mechanosensors on elytra and hind wings, or the area of Lehr’s field on elytra, evolve as a function of allometry, we regressed body length and absolute wing size on these response variables. We also examined whether flight investment (relative wing size) influenced the response variables. Species-level mean values were used in these analyses.

The regression analyses were performed using SLOUCH^71^, which models adaptive evolution of a trait along a phylogeny. In its most simple implementation, SLOUCH is run without predictors and returns a parameter value (half-life), which expresses the phylogenetic signal of the response variable. When the half-life parameter from an intercept-only model is zero, there is no effect of the ancestral state on the response variable, i.e., there is no phylogenetic signal. Increasing values of half-life estimates (which is expressed in units of tree length) indicate increasing influence of ancestral traits on current phenotypes (stronger phylogenetic signal), with trait evolution approaching a Brownian motion process as the half-life parameter approaches infinity. Such intercept-only models also serve as null models, with zero slopes, to which regression models are compared using δAICc.

When a predictor variable is added, SLOUCH utilizes an Ornstein–Uhlenbeck model and assumes that the trait (response variable) evolves towards a ‘primary’ optimum, which is defined as the average optimal phenotype reached when ancestral constraints have disappeared^72^. Any lag on the evolution towards this optimal phenotype is expressed via the half-life parameter (the time it takes to evolve halfway toward the evolutionary optimal state). Half-life is expressed as a proportion of the phylogeny’s length, hence we set the total length of our phylogeny to 1 in order to ease interpretation of the results. A half-life of zero means that changes in the response variable, as driven by the predictor, is instantaneous. A half-life of 1 means that evolving halfway to the evolutionary optimal state takes the time amounting to the entire species complex history. The larger the half-life estimate, the bigger the difference between the evolutionary and optimal regression slopes. The model also reports ‘stationary variance’, which gives the residual variance when stochastic equilibrium has been reached.

SLOUCH produces two effect sizes, or regression lines; an evolutionary regression, which shows the observed association between response and predictor, and an optimal regression line, which shows the predicted association in a scenario where there is no lag on the evolutionary process. This allows us to estimate the selective pressure imposed by the predictor variables, which in many cases can be shrouded by opposing selective pressures or physiological constraints.

SLOUCH regression models were compared to SLOUCH null models (without predictors) using δAICc (the difference in Akaike Information Criterion [lowercase c indicates a control for small sample size]). Following Burnham and Anderson^73^ we imposed a criterion of δAICc < − 2 constituting statistical significance.

Of the 28 species included in this study, one species—*Dactylipalpus* sp.—was a clear outlier in terms of sheer size. We opted to perform two sets of analyses, with and without *Dactylipalpus* sp.

### Methodological limitation

Reliable analysis of mechanosensors’ number on metathoracic wings and elytra of very small bark beetles is challenging, as it requires very precise and time-consuming preparation, especially hind wing straightening and precise, bubble-free coating with a thin layer of Euparal. This method of preparation hinders, however, the reliable count of mechanosensors at two small, most proximal CS fields located at the base of the radial vein (as this region is often damaged during wing straightening). We believe, however, that this limitation would not significantly affect the main findings of the study since we precisely analyzed the third largest, most distal, and also well-visible campaniform sensilla field located on the radial vein, and the area of closely located small sensing fields seems to be proportionally related to the area of the largest sensilla field. Our study only analyze mechanosensors located on dorsal side of wings and elytra. Although in some insect species mechanosensors have also been identified on the ventral side of the wing, their number seems to be marginal in the case of Coleoptera^8,10,13^.

Due to the cost, time-consuming nature, material availability, and invasiveness of the research procedures (the need to destroy the specimens using SEM), we limited the sample size to a minimum of 4 specimens (2 females and 2 males when possible) per species. To compensate for this limitation, we analyzed both the left and right elytra of each specimen. Although the number of mechanosensors seems to be relatively equal between specimens of the same species (Supplementary Information S1—Data S1), and the small observed differences are due to body size differences (see the “Results” section), we decided to include three different species of *Ips* and *Scolytus* varying in body size to check whether the within-species differences in MS number are smaller when compared to differences between small and large species from the same genera.

### Supplementary Information

Supplementary Information.

## Data Availability

Data used in the paper are available in the Supplementary Information S1—Data S1.
