# Supplementary material for: Evolution, types, and distribution of flight control devices on wings and elytra in bark beetles
Source: Sci Rep. 2024 Mar 24;14:6999. doi: 10.1038/s41598-024-57658-y (PMC10961309; doi:10.1038/s41598-024-57658-y)
Supplement: Supplementary file 1 — Supplementary Information. [file 41598_2024_57658_MOESM1_ESM.pdf]

Supplementary Information 1

Evolution, types, and distribution of flight control devices on wings and elytra in bark beetles

[1]Jakub Białkowski, [1]Robert Rossa, [1]Anna Ziemiakowicz, [2]Jostein Gohli, [3]Jakub Dymek, [1*]Jakub Goczał

[1]Department of Forest Ecosystems Protection, University of Agriculture in Krakow, 29 Listopada 54, 31-425 Krakow, Poland
[2]Division of Biotechnology and Plant Health, Norwegian Institute of Bioeconomy Research, Ås, Norway
[3]Department of Biology and Cell Imaging, Faculty of Biology, Institute of Zoology and Biomedical Research, Jagiellonian University, Krakow, Poland

[*]Author for correspondence (Tel.: +48 12 662 51 39; E-mail: jakub.goczal@urk.edu.pl).

Supplementary Table S1. Results from SLOUCH models where our measure of flight investment (relative wing area, i.e., residuals from a linear model where body length was regressed on wing area), body length and absolute wing area was regressed on mechanosensor response variables. Here, one outlier species (*Dactylipalpus* sp.) was included in the analysis. Abbreviations: MS – mechanosensors, WA – wing area.

Response	Predictor	n	Intercept only	Phylogenetic half life	Stationary variance	R^2	AICc	δAICc*	
Total number of MS on elytra	Relative WA	28	0.005	0.000	925	0.07	280	0.95	
	Body length	28		0.000	459	0.54	261	**-18.68**	
	Absolute WA	28		0.000	257	0.74	245	**-34.89**	
Lehr's field area on elytra	Relative WA	28	0.083	1208	3.E+10	0.31	566	-1.95	**
	Body length	28		0.201	9.E+06	0.56	549	**-19.09**	
	Absolute WA	28		0.019	2.E+06	0.89	510	**-58.41**	
Total number of MS on hind wings	Relative WA	28	0.723	571.432	1.E+07	0.15	362	-1.58	**
	Body length	28		0.047	7584	0.60	342	**-21.61**	
	Absolute WA	28		0.028	5583	0.72	333	**-30.68**	

* AICc values from SLOUCH models with relative wing size compared to null models. δAICc values < -2 are considered significant.

** Models with uncertain estimation (no clearly defined likelihood peak) of half life and stationary variance.

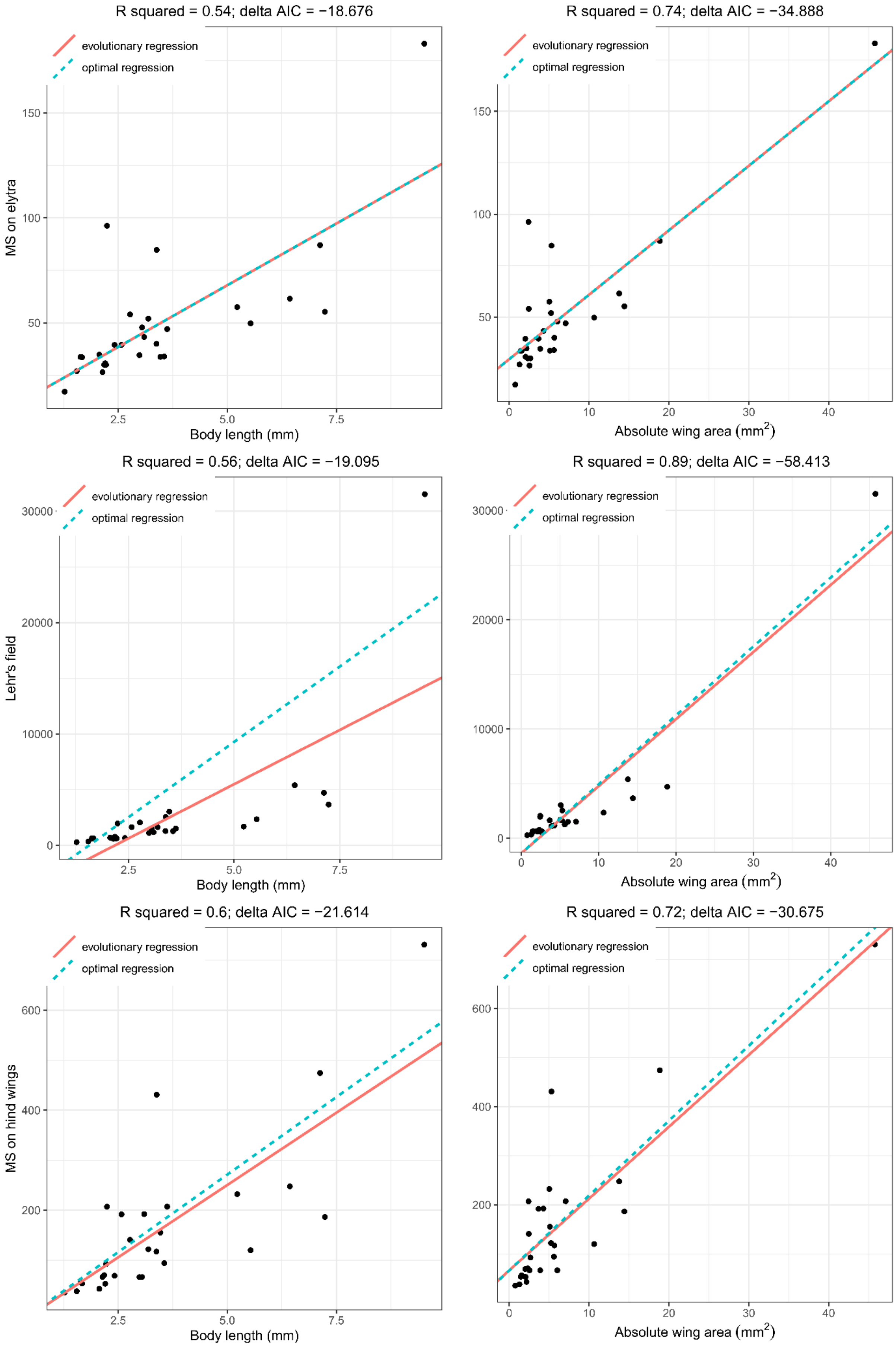
R squared = 0.54; delta AIC = −18.676
evolutionary regression
optimal regression
MS on elytra
Body length (mm)
R squared = 0.74; delta AIC = −34.888
evolutionary regression
optimal regression
Absolute wing area (mm2)
R squared = 0.56; delta AIC = −19.095
evolutionary regression
optimal regression
Lehr's field
Body length (mm)
R squared = 0.89; delta AIC = −58.413
evolutionary regression
optimal regression
Absolute wing area (mm2)
R squared = 0.6; delta AIC = −21.614
evolutionary regression
optimal regression
MS on hind wings
Body length (mm)
R squared = 0.72; delta AIC = −30.675
evolutionary regression
optimal regression
Absolute wing area (mm2)

Supplementary Fig. S1. Effect size of body length and absolute wing area regressed on the number of mechanosensors on elytra and hind wing, and the size of Lehr’s field. The evolutionary regression shows the observed association, while the optimal regression shows the effect size expected in a scenario with no phylogenetic inertia (evolutionary lag). One outlier species (*Dactylipalpus* sp.) was included in these analyses. The optimal regression is not shown for the regression of body length on mechanosensors on hind wings, due to uncertain estimation of the half-life parameter. R squared denotes the amount of variation in the response explained by the model, while delta AIC gives the significance level, with values < -2 being considered statistically significant.

Supplementary Table S2. Results from SLOUCH models where our measure of flight investment (relative wing area, i.e., residuals from a linear model where body length was regressed on wing area), body length and absolute wing area (WA) was regressed on (1) the number of campaniform sensilla (CS), (2) the number of trichoid sensilla (TS), and (3) the number of isolated trichoid sensilla (isolated CS). Intercept only half life indicated phylogenetic signal in response variable. Here, one outlier species (*Dactylipalpus* sp.) was removed from the analysis.

Response	Predictor	n	Intercept only half life	Phylogenetic half life	Stationary variance	Intercept	Evolutionary reg. slope	Optimal reg. slope	R2	AICc	?AICc*	
No. of CS	Relative WA	28	1.00	1436.7	22618653	171.8	41.6	172287.2	0.14	360	-1.54	**
	Body length	28		0.048	7183	-50.7	55.2	59.3	0.59	341	-20.74	
	Absolute WA	28		0.028	5196	48.6	14.1	14.7	0.71	331	-30.26	
No. of TS	Relative WA	28	0.22	0.332	112	20.6	3.8	6.5	0.07	217	0.94	
	Body length	28		5.508	388	9.6	2.2	35.7	0.32	210	-5.89	**
	Absolute WA	28		0.457	91	16.5	0.5	1.0	0.24	212	-4.36	
No.isolated TS	Relative WA	28	4054.63	600.0	5714	190.1	44.6	73618.3	0.00	151	2.73	**
	Body length	28		465.3	4388	2.8	0.1	104.9	0.01	151	2.56	**
	Absolute WA	28		1010	9592	3.1	0.0	1.5	0.00	151	2.74	**

* AICc values from SLOUCH models with relative wing size compared to null models. ?AICc values < -2 are considered significant.

** Models with uncertain estimation (no clearly defined likelihood peak) of half life and stationary variance.

Supplementary Table S3. Results from SLOUCH models where our measure of flight investment (relative wing area, i.e., residuals from a linear model where body length was regressed on wing area), body length and absolute wing area (WA) was regressed on (1) the number of campaniform sensilla (CS), (2) the number of trichoid sensilla (TS), and (3) the number of isolated trichoid sensilla (isolated CS). Intercept only half life indicated phylogenetic signal in response variable. Here, one outlier species (*Dactylipalpus* sp.) was included in the analysis.

Response	Predictor	n	Intercept only half life	Phylogenetic half life	Stationary variance	Intercept	Evolutionary reg. slope	Optimal reg. slope	R2	AICc	?AICc*	
No. of CS	Relative WA	27	0.16	155.1	1224372	153.3	37.4	16781.1	0.20	329	-1.23	**
	Body length	27		408.164	1395215	51.3	27.4	32252.6	0.46	318	-13.00	**
	Absolute WA	27		200.000	832933	112.2	7.3	4190.8	0.41	320	-10.39	**
No. of TS	Relative WA	27	0.22	0.328	112	20.2	3.5	6.0	0.06	209	1.30	
	Body length	27		6.728	491	9.6	2.2	43.9	0.30	204	-4.15	**
	Absolute WA	27		0.056	79	15.2	1.1	1.2	0.25	204	-4.27	
No. isolated TS	Relative WA	27	3927.70	1755.1	16327	3.3	0.0	-37.8	0.00	145	2.77	**
	Body length	27		514.3	4592	2.7	0.2	258.1	0.03	144	1.96	**
	Absolute WA	27		257	2327	3.0	0.0	31.7	0.02	144	2.15	

* AICc values from SLOUCH models with relative wing size compared to null models. ?AICc values < -2 are considered significant.

** Models with uncertain estimation (no clearly defined likelihood peak) of half life and stationary variance.

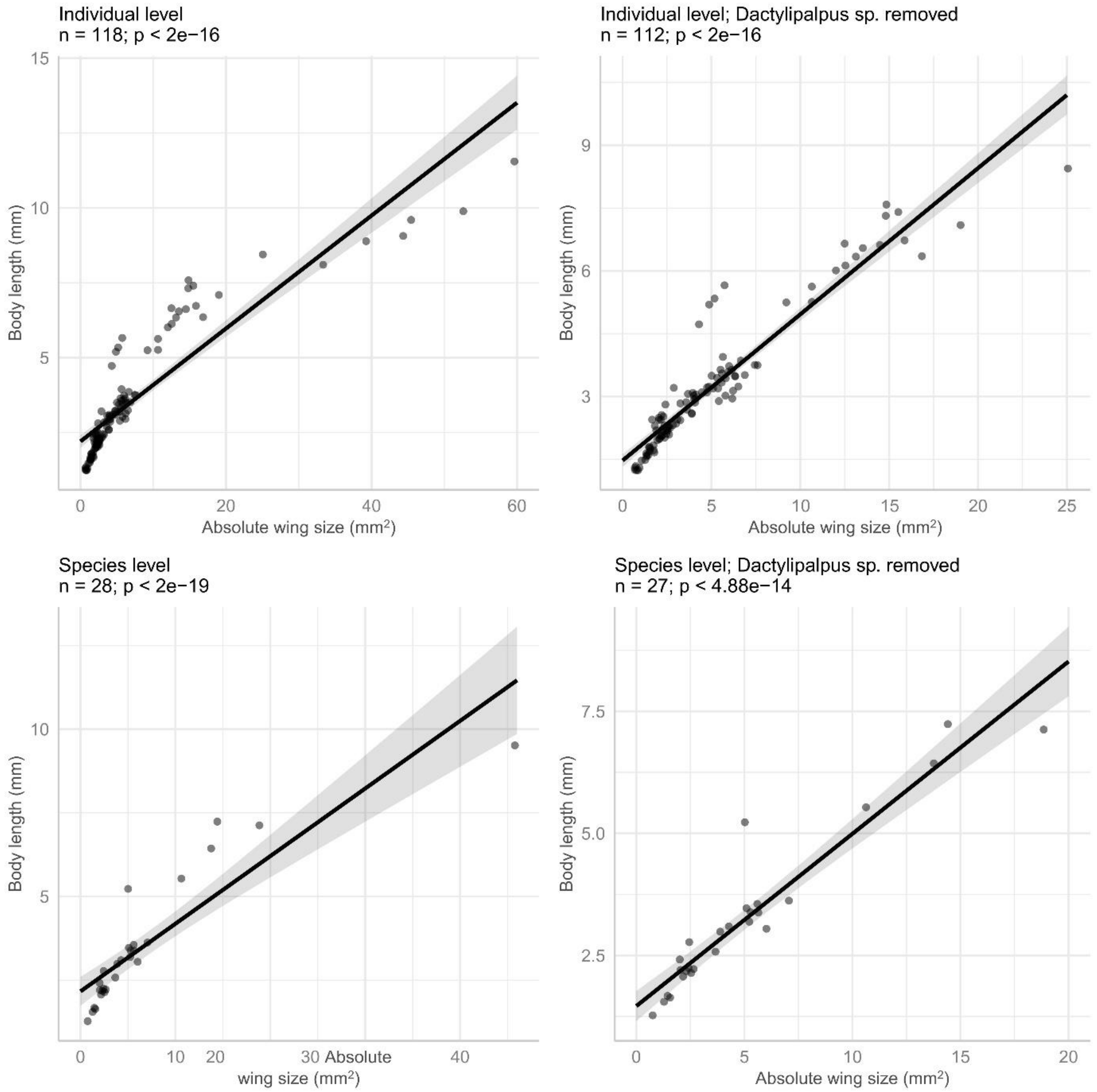

Supplementary Fig. S2. Linear regression between body length and absolute wing size in studied bark beetle species on individual level (upper graphs) and species level (lower graphs) with outlier species included (left side) and excluded (right side).

Supplementary Data S1.

Raw data used for analyses. Notice that males of *Anisandrus dispar* (marked with yellow) have reduced hind wings, and that in the case of *Dactylipalpus sp.* (marked with green) no males have been analyzed. Abbreviations: CS – campaniform sensilla, TS – trichoid sensilla.

ID	Species	Sex	Body length [mm]	Mean elytra area [mm2]	Mean single Lehr's field area [µm2]	Mean single wing area [mm2]	No. mechanosensors on hind wings	No. mechanosenors on elytra	No. CS on wings	No. TS on wings (including isolated)	No. isolated TS on wings
51	Anisandrus dispar	F	3.19	1.86	839.22	5.09	116	50	86	30	0
051a	Anisandrus dispar	M	2.39	1.17	471.14	0.07	35	36	31	4	0
051c	Anisandrus dispar	M	2.34	1.12	476.73	0.06	19	32	15	4	0
52	Anisandrus dispar	F	3.19	1.83	802.44	5.37	128	54	93	35	0
121	Cryphalus piceae	F	1.57	0.45	307.25	1.36	61	34	31	30	4
122	Cryphalus piceae	F	1.6	0.5	341.87	1.35	53	34	27	26	4
123	Cryphalus piceae	M	1.72	0.56	338.8	1.76	58	33	27	31	4
124	Cryphalus piceae	M	1.66	0.53	290.03	1.78	53	34	22	31	4
141	Crypturgus cinereus	F	1.24	0.33	145.87	0.87	37	18	17	20	4
142	Crypturgus cinereus	F	1.24	0.37	155.37	0.79	44	18	27	17	4
143	Crypturgus cinereus	M	1.25	0.25	141.57	0.69	39	16	20	19	4
144	Crypturgus cinereus	F	1.3	0.26	153.05	0.69	35	17	17	18	4
145	Crypturgus cinereus	M	1.34	0.26	133.78	0.74	21	17	13	8	2
147	Dactylipalpus sp.	F	11.55	11.96	19541.63	59.63	829	194	788	41	1
148	Dactylipalpus sp.	F	9.89	21.86	17799.71	52.61	744	186	707	37	0
149	Dactylipalpus sp.	F	9.6	17.81	11349.05	45.44	721	191	682	39	0
150	Dactylipalpus sp.	F	8.89	16.81	15884.72	39.25	758	183	719	39	0
151	Dactylipalpus sp.	F	8.1	14.34	14519.22	33.35	639	166	617	22	0
152	Dactylipalpus sp.	F	9.06	16.78	15501.11	44.35	695	178	666	29	0
153	Dactylotrypes longicollis	M	1.95	0.69	345.67	1.95	41	36	33	8	0
154	Dactylotrypes longicollis	M	2.05	0.77	342.88	2.21	41	37	36	5	0
155	Dactylotrypes longicollis	M	2.08	0.77	344.52	2.2	45	36	37	8	1
156	Dactylotrypes longicollis	F	2.19	0.86	389.8	2.44	42	34	36	6	0
157	Dactylotrypes longicollis	F	2.05	0.76	335.8	2.17	43	32	37	6	0
158	Dactylotrypes longicollis	F	2.09	0.78	332.66	2.04	45	34	36	9	2
71	Dendronocus micans	M	6.62	7.39	2060.86	14.48	436	84	383	53	15
72	Dendronocus micans	M	7.09	8.27	2149.47	19.02	492	84	445	47	12
73	Dendronocus micans	F	8.44	11.57	3575.39	25.06	558	96	502	56	21

74	Dendronocus micans	F	6.35	7.38	1650.66	16.84	411	84	385	26	12
16	Dryocetes alni	M	2.31	0.9	430.2	2.37	82	30	60	22	8
18	Dryocetes alni	F	2.31	0.9	430.2	2.82	67	30	51	16	6
19	Dryocetes alni	F	1.8	0.54	293.7	1.52	64	30	47	17	5
20	Dryocetes alni	M	2.29	0.84	396.13	2.58	68	30	52	16	5
116	Ernoporus tiliae	M	1.76	0.56	293.86	1.55	45	33	31	14	4
117	Ernoporus tiliae	M	1.84	0.67	361.59	1.75	63	33	47	16	4
118	Ernoporus tiliae	F	1.78	0.54	387.88	1.48	55	35	36	19	4
119	Ernoporus tiliae	F	1.69	0.52	293.62	1.53	57	34	39	18	5
120	Ernoporus tiliae	F	1.3	0.35	254.82	0.95	47	33	33	14	4
130	Gnathotrichus materiarius	M	3.05	1.42	644.83	4.12	69	36	57	12	0
131	Gnathotrichus materiarius	F	2.94	1.32	521.94	4.02	66	36	52	14	0
132	Gnathotrichus materiarius	F	3.03	1.3	553.92	4.01	64	33	52	12	0
133	Gnathotrichus materiarius	F	2.86	1.32	548.65	3.58	65	34	49	16	0
134	Gnathotrichus materiarius	M	3.06	1.34	509.21	3.68	68	34	55	13	0
31	Hylastes opacus	M	2.86	1.37	623.18	4.08	174	44	149	25	6
33	Hylastes opacus	F	3.49	1.65	599.68	5.01	224	42	198	26	4
34	Hylastes opacus	F	2.95	1.55	540.17	4.1	209	44	178	31	5
35	Hylastes opacus	M	3.09	1.5	600.89	3.95	162	43	133	29	6
81	Hylesinus fraxini	M	3.43	2.47	1495.6	5.8	450	90	416	34	11
82	Hylesinus fraxini	M	3.44	2.38	1297.9	5.32	431	92	399	32	9
84	Hylesinus fraxini	F	3.55	2.45	1174.68	5.59	532	78	497	35	10
115	Hylesinus fraxini	F	3.1	1.85	1138.54	4.46	311	79	278	33	12
135	Hylorgops palliatus	F	3.02	1.78	733.37	5.79	69	47	57	12	0
136	Hylorgops palliatus	F	3.14	1.96	784.8	6.22	66	48	52	14	0
137	Hylorgops palliatus	M	2.89	1.65	773.05	5.42	64	50	52	12	0
138	Hylorgops palliatus	M	2.95	1.87	759.33	6.17	65	45	49	16	0
140	Hylorgops palliatus	F	3.24	2.12	723.7	6.51	68	49	55	13	0
47	Ips acuminatus	M	3.73	1.69	709.19	6	100	40	83	17	3
48	Ips acuminatus	F	3.64	1.58	671.08	5.52	96	36	74	22	4
49	Ips acuminatus	F	3.64	1.84	623.63	6.14	99	30	76	23	4
50	Ips acuminatus	M	3.22	1.49	552.71	4.75	82	30	68	14	4
101	Ips cembrae	M	5.25	3.26	1123.03	9.21	108	46	78	30	4
102	Ips cembrae	M	6.01	4.39	1280.92	12.01	134	52	88	46	4
103	Ips cembrae	F	5.26	4.01	1234.06	10.65	114	51	78	36	4
104	Ips cembrae	F	5.62	3.98	1066.18	10.65	124	50	87	37	3
96	Ips sexdentatus	M	7.58	6.44	2094.62	14.84	204	58	167	37	4
97	Ips sexdentatus	M	7.4	6.52	1946.47	15.51	184	54	149	35	3

98	Ips sexdentatus	F	6.65	5.32	1703.3	12.5	169	56	135	34	4
99	Ips sexdentatus	F	7.31	6.2	1599.37	14.81	189	53	134	55	4
078a3	Orthotomicus laricis	F	3.1	1.47	601.96	4.74	122	34	98	24	4
078a5	Orthotomicus laricis	F	3.33	1.76	608.93	5.58	121	42	104	17	4
078b1	Orthotomicus laricis	M	3.59	1.83	685.57	5.94	112	42	92	20	4
078b2	Orthotomicus laricis	M	3.49	1.86	684.16	6.33	114	42	90	24	3
125	Phloesinus thujae	M	2.27	1.11	1005.76	2.6	204	98	172	32	4
126	Phloesinus thujae	M	2.28	1.12	972.36	2.53	204	95	170	34	4
127	Phloesinus thujae	F	2.19	0.99	1011.69	2.28	223	93	189	34	4
129	Phloesinus thujae	M	2.23	1.02	951.05	2.26	197	99	169	28	4
2	Pityogenes chalcographus	M	2.19	0.5	266.64	1.9	57	32	45	12	0
3	Pityogenes chalcographus	F	2.45	0.71	227.76	1.66	44	31	31	13	2
4	Pityogenes chalcographus	F	1.99	0.61	329.46	2.09	46	30	38	8	0
5	Pityogenes chalcographus	M	2.18	0.6	403.49	2.53	65	30	56	9	1
41	Pityokteines vorontzovi	M	2.21	0.7	320.41	2.48	65	26	51	14	2
42	Pityokteines vorontzovi	M	2.02	0.63	319.01	2.37	67	26	52	15	2
43	Pityokteines vorontzovi	F	2.25	0.75	275.99	2.68	68	26	53	15	3
44	Pityokteines vorontzovi	F	2.09	0.66	263.52	2.62	66	28	51	15	2
21	Pityophthorus pityographus	M	1.65	0.4	189.56	1.35	42	30	32	10	2
24	Pityophthorus pityographus	F	1.49	0.36	173.45	1.28	44	26	34	10	2
25	Pityophthorus pityographus	F	1.47	0.34	168.34	1.07	35	24	27	8	2
114	Pityophthorus pityographus	M	1.6	0.42	182.23	1.45	31	28	22	9	2
61	Platypus cylindricus	M	5.19	2.46	913.51	4.87	215	52	207	8	4
62	Platypus cylindricus	M	4.72	2.12	746.51	4.3	207	52	199	8	4
63	Platypus cylindricus	F	5.66	2.85	812.85	5.74	246	64	237	9	4
64	Platypus cylindricus	F	5.34	2.33	915.36	5.18	261	62	250	11	6
27	Polygraphus poligraphus	M	2.6	1.28	809.56	3.9	219	34	187	32	8
28	Polygraphus poligraphus	F	2.58	1.37	855.76	3.9	175	36	152	23	7
29	Polygraphus poligraphus	F	2.43	1.05	744.1	3.25	181	48	156	25	7
30	Polygraphus poligraphus	M	2.69	1.27	879.52	3.62	192	40	163	29	7
86	Scolytus ratzeburgii	M	6.34	5.22	2294	13.13	230	61	226	4	0
87	Scolytus ratzeburgii	M	6.54	5.45	2447.97	13.52	228	60	223	5	0
088a	Scolytus ratzeburgii	F	6.73	6.18	3364.02	15.87	294	66	287	7	0
89	Scolytus ratzeburgii	F	6.13	5.34	2704.65	12.54	239	59	230	9	0
68	Scolytus intricatus	F	2.84	1.25	1203.88	3.25	110	32	100	10	0
69	Scolytus intricatus	F	3.23	1.78	1486.65	4.85	182	32	162	20	0
111	Scolytus intricatus	M	3.86	2.39	1877.2	6.65	156	36	147	9	0

112	Scolytus intricatus	M	3.94	2.02	1506.67	5.65	173	35	162	11	0
91	Scolytus rugulosus	M	2.56	1.07	1136.48	2.2	129	50	122	7	0
92	Scolytus rugulosus	M	2.81	0.97	944.29	2.41	133	56	121	12	0
93	Scolytus rugulosus	F	3.21	1.27	1064.19	2.88	153	56	139	14	0
94	Scolytus rugulosus	F	2.52	0.98	980.74	2.3	148	54	135	13	0
12	Taphrorychus bicolor	M	2.35	0.87	315.59	3.01	106	30	91	15	0
13	Taphrorychus bicolor	F	2.11	0.76	283.1	2.47	86	30	72	14	1
14	Taphrorychus bicolor	F	2.45	0.84	352.17	3.08	99	30	86	13	0
15	Taphrorychus bicolor	M	1.98	0.69	321.27	2.06	79	30	68	11	0
058a	Trypodendron domesticum	F	3.51	2.21	838.56	6.88	184	53	158	26	4
59	Trypodendron domesticum	F	3.75	2.17	656.94	7.44	216	44	187	29	5
60	Trypodendron domesticum	M	3.48	2.18	649.47	6.34	194	44	160	34	4
113	Trypodendron domesticum	M	3.75	2.2	897.71	7.6	235	47	210	25	4
106	Xyleborinus sexesenii	M	2.44	0.79	332.92	2.15	64	39	54	10	1
107	Xyleborinus sexesenii	M	2.3	0.71	314.21	1.81	69	39	58	11	2
108	Xyleborinus sexesenii	F	2.49	0.81	344.38	2.04	77	40	64	13	2
109	Xyleborinus sexesenii	F	2.44	0.77	341.78	2.05	66	40	56	10	2
